# Frailty-associated factors among Brazilian community-dwelling elderly people: longitudinal study

**DOI:** 10.1590/1516-3180.2019.0179160919

**Published:** 2020-01-13

**Authors:** Maycon Sousa Pegorari, Darlene Mara dos Santos Tavares

**Affiliations:** I PhD. Physiotherapist and Adjunct Professor, Physiotherapy Course, Universidade Federal do Amapá (UNIFAP), Macapá (AP), Brazil.; II PhD. Nurse and Associate Professor, Department of Nursing Education and Community Health Nursing Graduate Program, Universidade Federal do Triângulo Mineiro (UFTM), Uberaba (MG), Brazil.

**Keywords:** Frail elderly, Longitudinal studies, Health services for the aged, Urban population, Primary prevention, Frailty assessment, Frailty syndrome, Frailty phenotype

## Abstract

**BACKGROUND:**

Frailty among elderly people is associated with negative health outcomes. Through gaining better understanding of this syndrome over different time periods, healthcare actions that take predictive factors into consideration may be facilitated.

**OBJECTIVE:**

To identify factors associated with frailty syndrome among community-dwelling elderly people over a two-year follow-up.

**DESIGN AND SETTING:**

Longitudinal study on elderly people living in Uberaba (MG), Brazil.

Methods: Elderly individuals were selected through multiple-stage conglomerate sampling from a national database. Participants were interviewed and evaluated in 2014 and again in 2016. Predictors were considered at the baseline, and frailty categories (frail, pre-frail or non-frail) at the follow-up. Frailty was identified based on the Fried criteria. Associations with socioeconomic factors, health status and physical performance were investigated using multinomial logistic regression.

**RESULTS:**

353 individuals participated in both assessments. The final model showed that age over 80 years was predictive of both pre-frailty and frailty (odds ratio, OR 4.92; 95% confidence interval, CI: 1.57-15.38; OR 8.64; 95% CI: 2.05-36.35, respectively), while dependency regarding basic activities of daily living (OR 3.66; 95% CI: 1.22-11.02) and poor lower-limb physical performance (OR 7.87; 95% CI: 1.97-31.39) predicted frailty. A one-unit increased score for advanced activities of daily living decreased the frailty rate by 15% (OR 0.85; 95% CI: 0.74-0.99).

**CONCLUSION:**

Age over 80 years was predictive of pre-frailty and frailty, while dependency in basic activities of daily living and poor physical performance predicted frailty. A one-unit increased score for advanced activities of daily living decreased the frailty rate by 15%.

## INTRODUCTION

Frailty among elderly people is considered to be a priority within public health. One reason for this is that presence of this syndrome predicts occurrences of adverse events that threaten the long-term sustainability of healthcare actions and systems. Moreover, frailty presents a negative influence on elderly people’s quality of life.^1^

Physical frailty is “a medical syndrome with multiple causes and contributing factors” that is characterized by impairment of “strength, endurance and physiological functions”, thus leading to “greater individual vulnerability in developing functional dependency and/or death”.^2^ From an operational point of view, the two measurements of frailty that have been most used (with high validity and reliability) are Fried’s frailty phenotype and Rockwood and Mitnitski’s frailty index.^3^

In a systematic review, frailty was found to be associated with several sociodemographic, physical, biological, lifestyle and psychological factors.^4^ Moreover, some risk factors for frailty were identified, such as advanced age, female gender, black race, lower income, lower educational level, cardiovascular diseases, multimorbidity, functional impairment, poor self-rated health, depressive symptoms, cognitive impairment, obesity, undernutrition, smoking and alcohol use.^5^

In Brazil, however, the available evidence is only recent and there is a lack of longitudinal studies analyzing the factors that determine frailty.^6^^,^^7^ In a study on 207 community-dwelling elderly people who were followed up for 12 months, the factors associated with frailty that predicted worsening of frailty status were histories of cancer, urinary incontinence and reduced capacity to perform advanced activities of daily living.^6^ Another one-year investigation conducted among 129 elderly people after hospital discharge did not identify any variables that were predictive of change (improvement or worsening) to frailty condition.^7^

Feng et al.^5^ considered that it was essential to determine the factors associated with frailty when developing interventions to prevent or reduce the frailty-associated burden among community-dwelling elderly people.

## OBJECTIVE

Given the low number of studies within the elderly population of Brazil and the need to understand the factors that determine frailty, the aim of this study was to identify frailty-associated factors among community-dwelling elderly people over a two-year follow-up.

## METHODS

### Ethics

This study was approved (protocol no. 493,211, dated December 13, 2013, and protocol no. 573,833, dated March 28, 2014) by the human-research ethics committee of the Federal University of the Triângulo Mineiro (Universidade Federal do Triângulo Mineiro, UFTM).

### Study design, participants and sample size

This was a longitudinal study, conducted among elderly people living in the urban area of Uberaba, state of Minas Gerais (MG), over a two-year follow-up (2014-2016). Uberaba is the main municipality of the area known as the “Triângulo Sul” of Minas Gerais, which is composed of 27 municipalities in the Triângulo Mineiro region of this state. In 2010, the estimated population of Uberaba was 328,272 citizens, its human development index (HDI) was 0.772 and life expectancy was 75.7 years.^8^ According to data from the Brazilian Institute for Geography and Statistics (Instituto Brasileiro de Geografia e Estatística, IBGE), Uberaba had an elderly population (i.e. of age greater than or equal to 60 years) of 37,365 people in 2010, which represented 12.62% of the total population.^8^

Population definition was done using a multiple-stage conglomerate sampling process. This process took into consideration the sectors defined by the Brazilian National Household Survey, with information from neighborhoods and streets that was made available by IBGE. Random household selection was conducted to identify elderly people in their homes.

The sample for the present study was composed of individuals who met the following inclusion criteria: (a) they participated at both times (2014 and 2016); (b) they did not present any cognitive deficit, as identified using the translated and validated Brazilian version of the Mini-Mental State Examination (MMSE), with cutoff points defined according to their educational level;^9^ (c) they were able to walk, with or without the use of walking aids (cane, crutches or walkers); and (d) they agreed to participate in the survey through signing a free and informed consent statement. Participants were excluded in the following situations: (a) inability to reach the participant, even after three attempts; (b) moving to another city; (c) occurrence of hospitalization at the time of the visit; and (d) presence of diseases that prevented the assessments. In the 2014 baseline assessment, 710 elderly people were interviewed.

**Figure 1.**
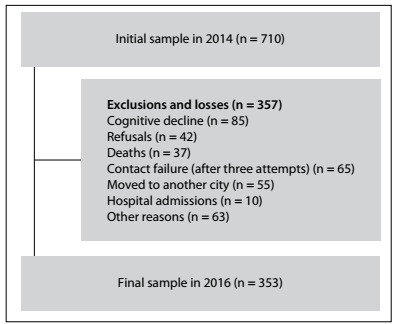
Study sample flow diagram.

In 2016, attempts were made to reach all the elderly people who had participated in the first stage of the survey (n = 710), in their homes. After the eligibility criteria and the losses had been taken into consideration (detailed in Figure 1; other reasons could be insufficient address or incomplete data), 353 elderly people were considered in the present investigation. Thus, these 353 individuals were evaluated both in 2014 and in 2016.

Because of the possibility of reading and comprehension problems, the interviews with the elderly people were conducted face-to-face in their homes. Therefore, interviewers (who were undergraduate and postgraduate students) were selected and trained regarding ethical issues within research and, additionally, they were accompanied by field supervisors (senior researchers).

### Dependent variable

The presence of frailty syndrome was investigated using the five items that Fried et al. described as components of the frailty phenotype.^10^ These were the following: (1) Presence of non-intentional weight loss, as assessed through the question “In the past year, did you lose 4.5 kg without intention?”; (2) Muscle strength loss verified based on handgrip strength, using a manual hydraulic dynamometer; the mean value from three measurements was obtained and the cutoff points proposed by Fried et al.^10^ were used; (3) Self-reported exhaustion and/or fatigue, as measured through two questions: “Did you feel that you had to make an effort to take care of your habitual tasks?” and “Were you unable to move forward with your things?”; (4) Presentation of slow walking speed, considering the time (in seconds) that was taken to walk a distance of 4.6 m, with the cutoff points as proposed by Fried et al.;^10^ and (5) Poor physical activity level, as ascertained using the long version of the International Physical Activity Questionnaire (IPAQ). Elderly people presenting three or more of these items were classified as frail; those with one or two of these items were classified as pre-frail; and those with none of these items were considered to be robust or non-frail.^10^ A detailed description of the components can be accessed in previous publications.^10^^,^^11^^,^^12^

### Exploratory variables

The following were considered to be exploratory (independent) variables:

1. Socioeconomic characteristics - age range in years (60 to 69, 70 to 79 or 80 or over), sex (male or female), marital status (with or without a companion), living arrangements (alone or with company), schooling in years (none, 1 to 4 or 5 or more) and individual monthly income in minimum wages (no income, less than or equal to 1 minimum wage, or 2 or more minimum wages);
2. Clinical health indicators - number of diseases, number of regular medications, health self-perception (very poor, poor, fair, good or very good), hospital admissions in the past 12 months (yes or no) and falls in the past 12 months (yes or no);
3. Functional incapacity - measured using patient-reported outcomes such as the Katz scale^13^ for basic activities of daily living (BADL); the Lawton and Brody scale for instrumental activities of daily living (IADL),^14^ categorized as dependent (total or partial dependency) or independent (without incapacity for BADL and IADL); and 13 questions of a social nature for advanced activities of daily living (AADL),^15^ in which the response alternatives were “never did”, “stopped doing” or “still doing”, with scoring in the range of 1-3 points, a minimum score of 13 points and a maximum of 39 points;
4. Fear of falling - measured using the Falls Efficacy Scale International - Brazil (FES-I Brazil), which was analyzed as a continuous variable, with scores ranging from 16 to 64;^16^ and
5. Physical performance - assessed using the Brazilian version of the Short Physical Performance Battery (SPPB), which was categorized as follows: 0-3 points, very poor performance; 4-6 points, poor performance; 7-9 points, moderate performance; and 10-12 points, good performance.^17^

### Statistical analysis

Statistical analysis was done using the absolute and percentage frequency distribution for categorical variables and central trend (mean) and dispersion (standard deviation) measurements for quantitative variables. Univariate and multivariate analyses were done using logistic multinomial regression analysis, in order to investigate associations between the exploratory variables and the dependent variable (frailty status). Thus, the exploratory variables (predictors) were obtained from the baseline (2014) and the frailty status (frail, pre-frail or non-frail) was obtained from the follow-up assessment (2016). The variables of interest were chosen in accordance with the criterion established (P < 0.20) and were included in the multivariate regression model. Predictors associated with pre-frailty and frailty were identified using odds ratios, through multinomial logistic regression, considering a significance level of 5% (P < 0.05) and a 95% confidence interval (CI). The data were analyzed using the Statistical Package for the Social Sciences (SPSS), version 21.0.

## RESULTS

In 2014, the majority of the 353 elderly people who were interviewed were women, in the age range of 60-69 years, and were living with a companion. Table 1 presents the distribution of the socioeconomic variables according to frailty status at the baseline.

**Table 1.** Socioeconomic, clinical and health variable distribution among the elderly people, according to the condition of frailty at the baseline. Uberaba (MG), Brazil, 2014 (n = 353)

Variables	Frailty syndrome							
	Frail (n = 34)		Pre-frail (n = 196)		Non-frail (n = 123)		Total	
	n	%	n	%	n	%	n	%
Age range (in years)								
60-69	12	35.3	92	46.9	68	55.3	172	48.7
70-79	12	35.3	78	39.8	40	32.5	130	36.8
80 or over	10	29.4	26	13.3	15	12.2	51	14.4
Sex								
Male	9	26.5	60	30.6	51	41.5	120	34.0
Female	25	73.5	136	69.4	72	58.5	233	66.0
Marital status								
With a companion	15	44.1	88	44.9	65	52.8	168	47.6
Without a companion	19	55.9	108	55.1	58	47.2	185	52.4
Living arrangements								
Alone	5	14.7	43	21.9	27	22	75	21,2
Accompanied	29	85.3	153	78.1	96	78	278	78.8
Educational level (in years)								
None	9	26.5	34	17.3	17	13.8	60	17.0
1-4	20	58.8	100	51	65	52.8	185	52.4
5 or more	5	14.7	62	31.6	41	33.3	108	30.6
Income								
No income	-	-	22	11.2	12	9.8	34	9.6
1 minimum wage* or lower	29	85.3	82	41.8	50	40.7	161	45.6
2 or more minimum wages	5	14.7	92	46.9	61	49.6	158	44.8
Health perception								
Positive	3	8.8	75	38.3	74	60,2	152	43.1
Negative	31	91.2	121	61.7	49	39.8	201	56.9
Hospital admission (past year)								
Yes	11	32.4	32	16.3	15	12.2	58	16.4
No	23	67.6	164	83.7	108	87.8	295	83.6
Falls								
Yes	16	47.1	46	23.5	24	19.5	86	24.4
No	18	52.9	150	76.5	99	80.5	267	75.6
Number of diseases (mean ± SD)	7.97 ± 3.91		6.14 ± 3.42		4.69 ± 3.23		5.81 ± 3.53	
Number of medications (mean ± SD)	4.97 ± 3.11		3.61 ± 2.63		2.48 ± 2.37		3.34 ± 2.69	
BADL								
Dependent	15	44.1	35	17.9	13	10.6	63	17.8
Independent	19	55.9	161	82.1	110	89.4	290	82.2
IADL								
Dependent	30	88.2	103	52.6	58	47.2	191	54.1
Independent	4	11.8	93	47.4	65	52.8	162	45.9
AADL (mean ± SD)	25.2 ± 2.86		27.53 ± 2.92		28.1 ± 3.46		27.51 ± 3.20	
FES-I-Brazil (mean ± SD)	35.26 ± 14.91		26.14 ± 12.33		22.88 ± 9.83		25.88 ± 12.26	
SPPB (mean ± SD)	5.21 ± 2.23		8.61±2.12		9.93 ± 1.63		8.74 ± 2.36	

BADL = basic activities of daily living; IADL = instrumental activities of daily living; AADL = advanced activities of daily living; FES-I-Brazil = Falls Efficacy Scale International - Brazil; SPPB = Short Physical Performance Battery; SD = standard deviation.

Table 2 presents the univariate analysis on the frailty-associated factors during the follow-up. Predictors were considered at the baseline, and frailty categories (frail, pre-frail or non-frail) in the follow-up assessment.

**Table 2.** Socioeconomic, clinical and health variables associated with the condition of frailty, using univariate analysis. Uberaba (MG), Brazil, 2014-2016 (n = 353)

Variables	Frailty syndrome					
	Pre-frail			Frail		
	OR	95% CI	P	OR	95% CI	P
Age range (in years)						
60-69	1			1		
70-79	1.68	1.01-2.78	0.045	2.17	0.97-4.85	0.058
80 or over	6.47	2.19-19.13	0.001	16.10	4.64-55.84	< 0.001
Sex						
Male	1			1		
Female	0.86	0.53-1.42	0.567	0.98	0.47-2.04	0.952
Marital status						
With a companion	1			1		
Without a companion	0.88	0.55-1.41	0.601	1.01	0.50-2.01	0.980
Living arrangements						
Alone	0.92	0.52-1.63	0.788	0.98	0.43-2.27	0.984
Accompanied	1			1		
Educational level (in years)						
None	2.04	0.94-4.41	0.071	2.71	0.99-7.44	0.052
1-4	1.22	0.72-2.05	0.456	0.92	0.41-2.04	0.837
5 or more	1			1		
Income						
No income	0.86	0.39-1.89	0.714	0.44	0.09-2.16	0.315
1 minimum wage or lower	1.22	0.74-2.01	0.429	1.71	0.83-3.52	0.147
2 or more minimum wages	1			1		
Health perception						
Positive	1			1		
Negative	1.38	0.86-2.21	0.177	2.58	1.23-5.44	0.012
Hospital admission (past year)						
Yes	1.25	0.65-2.43	0.503	1.74	0.72-4.23	0.221
No	1			1		
Falls						
Yes	1.71	0.94-3.11	0.080	3.25	1.49-7.08	0.003
No	1			1		
Number of diseases (mean ± SD)	1.05	0.98-1.13	0.133	1.15	1.05-1.27	0.004
Number of medications (mean ± SD)	1.05	0.96-1.15	0.271	1.19	1.06-1.36	0.004
BADL						
Dependent	1.62	0.79-3.28	0.183	5.19	2.24-12.07	< 0.001
Independent	1			1		
IADL						
Dependent	2.26	1.39-3.64	0.001	4.05	1.91-8.56	< 0.001
Independent	1			1		
AADL	0.94	0.87-1.01	0.111	0.78	0.71-0.89	< 0.001
FES-I-Brazil (mean ± SD)	1.01	0.99-1.03	0.246	1.04	1.01-1.07	0.003
Physical performance (SPPB)						
Very poor	3.82	0.44-33.52	0.226	56.87	6.18-523.79	< 0.001
Poor	2.80	1.07-7.31	0.035	23.02	7.03-75.33	< 0.001
Moderate	1.78	1.07-2.94	0.025	3.07	1.18-8.01	0.022
Good	1			1		

OR = odds ratio; 95% CI = 95% confidence interval; P < 0.20; 1 = reference category - non-frail group; BADL = basic activities of daily living; IADL = instrumental activities of daily living; AADL = advanced activities of daily living; FES-I-Brazil = Falls Efficacy Scale International - Brazil; SPPB = Short Physical Performance Battery.

The variables included in the multivariate model of the multinomial logistic regression are presented in Table 3. Age in the range of 80 years or over was a predictor of both frailty (OR = 8.64; 95% CI: 2.05-36.35) and pre-frailty (OR = 4.92; CI: 1.57-15.38), while dependency in basic activities of daily living (OR = 3.66; 95% CI: 1.22-11.02) and poor physical performance (OR = 7.87; 95% CI: 1.97-31.39) were predictors of frailty. Additionally, the results indicated that an increase of one point in the score for advanced activities of daily living decreased the rate of occurrence of the condition of frailty among these elderly individuals by 15% (OR = 0.85; 95% CI: 0.74-0.99) (Table 3).

**Table 3.** Final multinomial logistic regression model including the variables associated with the condition of frailty in a population of community-dwelling elderly people. Uberaba (MG), Brazil, 2014-2016 (n = 353)

Variables	Frailty syndrome					
	Pre-frail			Frail		
	OR	95% CI	P	OR	95% CI	P
Age range (in years)						
60-69	1			1		
70-79	-			-		
80 or over	4.92	1.57-15.38	0.006	8.64	2.05-36.35	0.003
BADL						
Dependent	-			3.66	1.22-11.02	0.021
Independent	1			1		
AADL	-			0.85	0.74-0.99	0.037
Physical performance (SPPB)						
Very poor	-			-		
Poor	­­­­­-			7.87	1.97-31.39	0.003
Moderate	-			-		
Good	1			1		

OR = odds ratio; 95% CI = 95% confidence interval; P < 0.05; 1 = reference category - non-frail group; BADL = basic activities of daily living; AADL = advanced activities of daily living; SPPB = Short Physical Performance Battery.

## DISCUSSION

The present study identified frailty predictors over a two-year follow-up period. These included advanced age, dependency relating to BADL and poor physical performance. On the other hand, ability to perform AADL provided a protective effect.

The results indicated that advanced age (80 years or over) was an independent predictor for both pre-frailty and frailty. Other investigations have also found that age was a frailty marker,^18^^,^^19^^,^^20^ including two systematic reviews.^4^^,^^5^

Age is an important indicator of the association between frailty categories and mortality.^21^ A systematic review indicated that the numbers of pre-frail and frail elderly people become greater at advanced ages, which suggests that frailty is a progressive condition and, hence, that it may appear more frequently among elderly people older than 80 years.^5^ Moreover, Fulop et al.^22^ discussed the existence of common but non-identical pathways of frailty and aging; they suggested that the characteristics of frailty syndrome were more accentuated than those of regular ageing. Thus, all individuals older than 70 years would need to be screened for frailty syndrome, in order to improve the management of individuals with this condition.^2^^,^^23^

The association of BADL dependency as a frailty predictor seen in the present study is divergent from the findings of other Brazilian studies.^6^^,^^7^ Nevertheless, an investigation in Italy, with a 4.4-year follow-up, found that worsening of the condition presented by non-frail individuals was associated with dependency in relation to activities of daily living.^18^ Furthermore, according to Fried et al.,^24^ functional incapacity may cause difficulty in accessing healthcare services or actions from healthcare professionals, which would lead to increases in unrecognized and unaddressed healthcare needs.^24^ Thus, implementation of monitoring actions and control over functional incapacity factors are strategies not only for maintaining functional capacity among elderly people,^25^^-^^26^ but also for prevention of consequent conditions of frailty.

The present study found that an increase of one unit in the AADL score may have a protective effect against occurrences of frailty. These results are corroborated by an investigation among Brazilian elderly people that identified that the chance that frailty would worsen within 12 months was smaller (20%) when the elderly individual was categorized as “still doing” an AADL.^6^

AADLs are complex activities involving social interaction, such as work or participation in community groups, meetings, cultural events, trips and other activities.^15^ Hence, they represent integrity of physical function, social function and performance in social roles.^27^ In addition, they are predictors of frailty.^28^ Therefore, elderly people with active social networks are likely to be less frail than those with less social engagement.^29^ Moreover, social participation and factors such as security, strong social cohesion and neighborhood belongingness^29^ are protective and provide balance in community frailty levels.^30^

Another frailty predictor is poor physical performance (4-6 points), as assessed using the SPPB. An Italian study with a mean follow-up period of 4.4 years found that poor physical performance (score lower than 8 points) was significantly associated with increased risk of becoming frail and with worsening frailty status.^18^

Previous cross-sectional studies identified the feasibility of using the SPPB to detect frailty among elderly people (score lower than 9 points),^31^ including detection of early signs of frailty before occurrence of slow walking speed among very old people (score of 8 points).^32^ Cesari et al.^33^^,^^34^ highlighted that the SPPB identified elderly people with greater vulnerability to stressors and elevated risk of negative health-related events, which are matters related to frailty syndrome. Therefore, these findings may explain the results from the present study.

The SPPB provides a simple measurement of physical performance that is easy to carry out, without any need for special equipment or extensive training for evaluators.^32^ Furthermore, it is one of the clinical tools most used for identifying frailty.^35^ Additionally, it provides a viable and objective definition for the complex concept of frailty, both in clinical practice and in research.^33^^,^^34^

Among the limitations of the present study, there were considerable losses of follow-up. A further limitation was that absence of cognitive decline was considered to be an inclusion criterion in the present study, given that presence of cognitive decline could have interfered with comprehension of the variables analyzed (especially considering the self-reported nature of some of the data). Moreover, it needs to be acknowledged that a relationship between frailty and cognitive decline exists.

In the light of the results from the present study and the fact that frailty is a highly prevalent syndrome in aging populations,^1^ it is imperative to identify and manage this condition properly.^23^ In this regard, knowledge of frailty-associated factors and the complexity of their determinants aids construction of early preventive and intervention actions.^5^^,^^12^

## CONCLUSION

Being 80 years of age or older was a predictor for conditions of pre-frailty and frailty, while dependency in basic activities of daily living and poor physical performance were predictive of frailty. An increase of one unit in the score for advanced activities of daily living decreased the rate of occurrence of the condition of frailty among these elderly people by 15%
